# Cottonseed Oil in Diets for Broilers in the Pre-Starter and Starter Phases

**DOI:** 10.1371/journal.pone.0147695

**Published:** 2016-01-25

**Authors:** Vânia Batista de Sousa Lima, Leilane Rocha Barros Dourado, Luciana Pereira Machado, Daniel Biagiotti, Stélio Bezerra Pinheiro de Lima, Guilherme José Bolzani de Campos Ferreira, Leonardo Atta Farias, Francinete Alves de Sousa, Raian Malta Acácio, Danilo Rodrigo Silva e Silva

**Affiliations:** 1 Animal Nutrition Laboratory, Federal University of Piauí, Teresina, Piauí, Brazil; 2 Department of Animal Science, Federal University of Piauí, Bom Jesus, Piauí, Brazil; 3 Department of Veterinary Science, Federal University of Fronteira do Sul, Realeza, Paraná, Brazil; 4 Technical College, Federal University of Piauí, Bom Jesus, Piauí, Brazil; 5 Department of Veterinary Science, Federal University of Piauí, Bom Jesus, Piauí, Brazil; 6 Federal University of Piauí, Bom Jesus, Piauí, Brazil; IPK, GERMANY

## Abstract

The objective of this study was to evaluate the effect of different levels of crude cottonseed oil in isoenergetic diets, with or without supplementation of ferrous sulfate, on performance variables, relative weight of organs, and blood parameters of broilers, and on the economic viability of diets in the periods from 1 to 7 and 1 to 21 days of age. A total of 600 male birds of the Ross line were distributed in a completely randomized design in a (4×2) factorial arrangement with eight treatments (0, 2, 4, and 6% cottonseed oil with and without ferrous sulfate), and five replicates. The following variables were studied: feed intake, weight gain, feed conversion, weight of organs, blood parameters, and yield of carcass and cuts at 21 days. No effects of the levels of cottonseed oil were found on the performance of animals aged 1 to 7 days, or on the relative weights of the organs. In this same period, the weight gain, and the relative weights of heart, liver, and intestine of the animals that received ferrous sulfate were decreased, and feed conversion was worsened. In the period from 1 to 21 days, weight gain increased linearly with the increase in the levels of cottonseed oil. Blood parameters were not influenced by the diets. Crude cottonseed oil can be utilized in diets for broilers in the periods from 1 to 7 and 1 to 21 days of age at up to 6% of inclusion, and supplementation with ferrous sulfate is unnecessary if the differences in metabolization of the cottonseed oil are considered, with and without, it during the diet formulation process.

## Introduction

Corn is one of the most commonly utilized raw materials in the formulation of diets for monogastric animals, as an energy food, together with soybean meal, the main protein ingredient. However, the price of these ingredients fluctuates, especially during off-season periods. Thus, it is necessary to know the chemical composition and the antinutritional factors of alternative ingredients that can substitute partially or totally the conventional feedstuffs, aiming mainly at reducing the feed costs, which represent around 70% of the expenses with production, without impairing animal performance [1].

Broiler lines have been improved and currently display accelerated growth, which makes them more demanding nutritionally. In view of the high energy requirement of these birds, lipids of animal origin, like beef tallow, or plant-derived, like the oils extracted from oilseeds, have been used as important energy sources in the formulation of broiler diets [2], so as to meet the energy requirements. Besides, lipids in diets improve the palatability; increase the aggregation of dietary particles; help the digestion and absorption of nutrients such as liposoluble vitamins; and are important sources of essential fatty acids like linoleic and linolenic acid [3].

Because of their nutritional composition, cotton by-products such as the meal and the cake are utilized as alternative protein sources in the nutrition of monogastrics. In the harvest, the seed cotton is obtained, consisting of the fiber and the seed itself, which contains the hulls, and where the shortest and well-adhered cellulose fibers, named lint, are located. The seed is rich in oil, containing 30 to 40% protein and 35% to 40% lipids; thus, it is one of the main raw materials for the edible oil industry [4].

The crude cottonseed oil is extracted from the seed basically in two ways: by mechanical pressing, or through the use of chemical extractants or solvents. This oil is usually dark in color due to the presence of pigments produced by glands present in the cotyledons and hypocotyl of the seeds. It contains mainly the palmitic, oleic, and linoleic acids, which correspond to approximately 24%, 22%, and 52%, respectively, although other acids can be found in lower amounts [5]. The cottonseed oil is also rich in vitamin E, an important natural antioxidant that provides it with greater oxidative stability [4].

Although the crude cottonseed oil has recognized nutritional properties, the biggest obstacle to its utilization in diets for monogastrics is the presence of a toxic polyphenolic pigment (C_30_H_30_O_8_) named gossypol, present exclusively inside glands distributed in the leaves, stems, roots, and especially seeds of cotton (*Gossypium* sp.). Gossypol can trigger undesirable effects in the body, greatly reducing the productive performance of animals [6]. In general, the signs of toxicity from gossypol involve loss of appetite; low weight gain; hypoprothrombinemia; diarrhea; reduction of hemoglobin, erythrocytes, and serum protein; edematous fluid in the abdominal cavity, lungs, and heart; degenerative diseases of the liver, spleen, and small intestine; discoloration of yolk; and decreased hatchability of eggs [7].

Research conducted with cotton by-products in diets for monogastrics has demonstrated a drop in animal performance rates caused by the high levels of inclusion of these ingredients. In a study conducted by Kakani et al. [8] evaluating the toxicity of gossypol in pullets and chickens, the authors observed reductions in feed intake during the second week of life of the birds as the main toxic effect of gossypol, which resulted in low weight gain.

In birds, the adverse effects of gossypol are related mainly to reduction of feed intake, egg production, egg weight, and body weight gain. However, in small amounts, gossypol does not influence the performance of the birds [9]. Because of the lack of information on the use of cottonseed oil in the feeding of broilers, the present study was conducted to evaluate the effect of crude cotton oil alone or in combination with ferrous sulfate supplementation in isoenergetic diets on performance, relative weight of organs, carcass yield, and blood parameters of broilers in the periods from 1 to 7 and 1 to 21 days of age.

## Materials and Methods

### Ethics Statement

This study was conducted in strict accordance with the recommendations of the Guide for the Care and Use of Laboratory Animals of the National Institutes of Health. The protocol was approved by the Ethics Committee in Animal Experimentation of the Federal University of Piauí (Piauí, Brazil) under the number 030/12.

### Birds and Housing

The experiment was conducted in the poultry farming section, at Federal University of Piauí, located in Bom Jesus-PI, Brazil. A total of 600 male Ross chickens were utilized in a completely randomized design in a 4 × 2 (four levels of oil, with or without ferrous sulfate) factorial arrangement with five replicates of 15 birds per experimental unit. Birds were housed in 2-m^2^ boxes containing bunk feeders and pendulum-activated drinkers, located in a masonry shed covered with ceramic tiles and with cement floor. The dividers between the boxes were made of flat wire mesh, and curtains were used to control the temperature and drafts. The four levels of cottonseed oil were 0, 2, 4, and 6%, considering the energy requirement of each phase recommended by Rostagno et al. [10]. Ferrous sulfate (Fe_2_(SO_4_)_3_) was included at the level of 0.1%, in accordance with Santos et al. [11].

### Diets and Feeding Program

The experimental diets were formulated to meet the nutritional requirements of the broilers in the studied phases, following Rostagno et al. [10] recommendations (Tables 1 and 2). The cottonseed oil utilized was obtained by the mechanical pressing method; it contained 1.2% gossypol and 9,120 kcal/kg gross energy. In the formulation, the composition of ingredients described in Rostagno et al. [10] was adopted, except for the cottonseed oil, in which the metabolizable energy content of 7,732 kcal/kg determined in a previous study was considered for the crude cottonseed oil without ferrous sulfate, and 8,270 kcal/kg for the cottonseed oil with ferrous sulfate.

**Table 1:** Centesimal composition of the experimental diets for broilers from 1 to 7 days of age.

	Treatment (Level of cottonseed oil)							
Ingredient (%)	0%	2%	4%	6%	0% + FS	2% + FS	4% + FS	6% + FS
Corn	59.960	57.060	51.908	46.755	59.960	56.702	51.191	45.679
Soybean meal 48%	34.862	35.354	36.229	37.103	34.862	35.415	36.350	37.286
Dicalcium phosphate	1.894	1.897	1.902	1.908	1.894	1.897	1.903	1.909
Limestone	0.845	0.841	0.834	0.828	0.845	0.840	0.833	0.827
Soybean oil	0.770	0.000	0.000	0.000	0.770	0.000	0.000	0.000
Common salt	0.506	0.508	0.510	0.512	0.506	0.508	0.510	0.512
Vit. min. supplement^1^	0.400	0.400	0.400	0.400	0.400	0.400	0.400	0.400
DL-methionine	0.347	0.349	0.354	0.358	0.347	0.350	0.354	0.359
L-lysine HCL	0.317	0.307	0.289	0.271	0.317	0.306	0.286	0.267
Inert^2^	0.100	1.284	3.575	5.866	0.000	1.483	4.072	6.661
Cottonseed oil	0.000	2.000	4.000	6.000	0.000	2.000	4.000	6.000
Ferrous sulfate	0.000	0.000	0.000	0.000	0.100	0.100	0.100	0.100
Total	100.0	100.0	100.0	100.0	100.0	100.0	100.0	100.0
	**Nutritional composition**							
Linoleic acid (%)	1.829	1.362	1.270	1.178	1.829	1.355	1.257	1.159
Calcium (%)	0.920	0.920	0.920	0.920	0.920	0.920	0.920	0.920
Chlorine (%)	0.355	0.354	0.353	0.351	0.355	0.354	0.352	0.351
Apparent metabolizable energy (Mcal/kg)^3^	2.925	2.925	2.925	2.925	2.925	2.925	2.925	2.925
Available phosphorus (%)	0.470	0.470	0.470	0.470	0.470	0.470	0.470	0.470
Digestible lysine (%)	1.304	1.304	1.304	1.304	1.304	1.304	1.304	1.304
Digestible met + cys. (%)	0.939	0.939	0.939	0.939	0.939	0.939	0.939	0.939
Crude fiber (%)	2.498	2.468	2.468	2.468	2.468	2.468	2.468	2.468
Potassium (%)	0.909	0.911	0.915	0.918	0.909	0.911	0.915	0.919
Crude protein (%)	22.000	22.000	22.000	22.000	22.000	22.000	22.000	22.000
Sodium (%)	0.220	0.220	0.220	0.220	0.220	0.220	0.220	0.220
Iron (mg/kg)^4^	188	188	188	188	388	388	388	388

FS, ferrous sulfate;

^1^Provided per kg of product: (Starter): folic acid—200.00 mg; biotin—10,000 mg; chloro-hydroxyquinoline—7,500.00 mg: zinc—17.50 g; vit. A—1,680,000.00 IU; vit. B1–436.50 mg; vit. B12–2,400.00 mg; vit. B2–1,200.00 mg; vit. B6–624.00 mg; vit. D3–400,000.00 IU; vit. E—3,500.00 mg; vit. K3–360.00 mg; niacin—8,400.00 mg; monensin sodium—25,000.00 mg; pantothenic acid—3,119.00 mg; choline chloride—80,710 mg; selenium -75.00 mg; ferrous sulfate—11,250 mg; manganese monoxide—18,740.00 mg; copper sulfate—1,996.00 mg; iodine—187.47 mg.

^2^Inert—sand;

^3^AME of cottonseed oil—7,732 kcal/kg and 8,270 kcal/kg with and without ferrous sulfate;

^4^Considering the iron content in corn, soybean meal, dicalcium phosphate, vitamin-mineral supplement, and ferrous sulfate.

**Table 2:** Centesimal composition of the experimental diets for broilers from 8 to 21 days of age.

	Treatment (Level of cottonseed oil)							
Ingredient (%)	0%	2%	4%	6%	0% + FS	2% + FS	4% + FS	6%+FS
Corn	65.334	62.110	56.958	51.805	65.230	61.752	56.241	50.729
Soybean meal 48%	30.023	30.570	31.444	32.319	30.057	30.631	31.566	32.501
Dicalcium phosphate	1.474	1.477	1.483	1.488	1.474	1.478	1.483	1.489
Limestone	0.994	0.990	0.983	0.977	0.993	0.989	0.982	0.975
Soybean oil	0.659	0.000	0.000	0.000	0.728	0.000	0.000	0.000
Common salt	0.480	0.482	0.484	0.486	0.481	0.482	0.484	0.487
Vit. min. supplement^1^	0.400	0.400	0.400	0.400	0.400	0.400	0.400	0.400
DL-methionine	0.271	0.274	0.278	0.283	0.271	0.274	0.279	0.284
L-lysine HCL	0.262	0.251	0.233	0.215	0.261	0.250	0.230	0.211
Inert^2^	0.100	1.443	3.734	6.024	0.000	1.641	4.231	6.820
Cottonseed oil	0.000	2.000	4.000	6.000	0.000	2.000	4.000	6.000
Ferrous sulfate	0.000	0.000	0.000	0.000	0.100	0.100	0.100	0.100
Total	100.0	100.0	100.0	100.0	100.0	100.0	100.0	100.0
	**Nutritional composition**							
Linoleic acid (%)	1.834	1.421	1.330	1.238	1.868	1.415	1.317	1.219
Calcium (%)	0.860	0.860	0.860	0.860	0.860	0.860	0.860	0.860
Chlorine (%)	0.340	0.340	0.340	0.340	0.340	0.339	0.338	0.337
Apparent metabolizable energy (Mcal/kg)^3^	2.980	2.980	2.980	2.980	2.980	2.980	2.980	2.980
Available phosphorus (%)	0.384	0.384	0.384	0.384	0.384	0.384	0.384	0.384
Digestible lysine (%)	1.141	1.141	1.141	1.141	1.141	1.141	1.141	1.141
Digestible met+cys. (%)	0.822	0.822	0.822	0.822	0.822	0.822	0.822	0.822
Crude fiber (%)	2.388	2.355	2.302	2.250	2.386	2.351	2.295	2.239
Potassium (%)	0.823	0.825	0.829	0.832	0.823	0.825	0.829	0.832
Crude protein (%)	20.000	20.000	20.000	20.000	20.000	20.000	20.000	20.000
Sodium (%)	0.210	0.210	0.210	0.210	0.210	0.210	0.210	0.210
Iron (mg/kg)^4^	165	165	165	165	365	365	365	365

FS, ferrous sulfate;

^1^Provided per kg of product: folic acid—200.00 mg; biotin—10,000 mg; chloro-hydroxyquinoline—7,500.00 mg: vit. A—1,680,000.00 IU; vit. B1–436.50 mg; vit. B12–2,400.00 mg; vit. B2–1,200.00 mg; vit. B6–624.00 mg; vit. D3–400,000.00 IU; vit. E—3,500.00 mg; vit. K3–360.00 mg; niacin—8,400.00 mg; monensin sodium—25,000.00 mg; pantothenic acid—3,119.00 mg; choline chloride—80,710 mg; selenium -75.00 mg; ferrous sulfate—11,250 mg; manganese monoxide—18,740.00 mg; copper sulfate—1,996.00 mg; iodine—187.47 mg.

^2^Inert—sand;

^3^AME of cottonseed oil—7,732 kcal/kg and 8,270 kcal/kg with and without ferrous sulfate;

^4^Considering the iron content in corn, soybean meal, dicalcium phosphate, vitamin-mineral supplement, and ferrous sulfate.

### Data and Sample Collection

Humidity and maximum and minimum temperatures within the shed were monitored by a thermo-hygrometer placed in the center of the shed at the height of the back of the birds. Readings were taken daily. Water was supplied ad libitum, and changed twice to prevent heating and fermentation. A continuous lighting program (natural + artificial light) was adopted, and diets were also supplied ad libitum.

At the end of the experimental phases, the following variables were calculated: feed intake (FC), obtained as the difference between the amount of feed supplied and the orts; weight gain (WG), determined as the difference between the weights of the birds at the end and start of the phase; and feed conversion (FC), calculated based on the feed intake and weight gain data in the period. The FI and average WG were calculated as a function of the number of birds per plot, and, in cases of mortality, they were defined as a function of the corrected number of bids as described by Sakomura & Rostagno [12].

At 7 and 21 days of age, one bird having weight close to the average weight of the experimental plot (in a total of five birds per treatment) was slaughtered by cervical dislocation after 6 h of feed deprivation, so the organs (heart, liver, gizzard+proventriculus, bursa, spleen, pancreas, and intestine) were removed and their relative weights were determined in relation to the live weight of the feed-deprived birds.

At 21 days of age, three birds having weight close to the average weight of the box were slaughtered after being deprived of feed for six hours, for the evaluation of the yields of carcass and cuts. After the feed-deprivation period, the birds were weighed individually to determine their live weight, and then they were slaughtered, bled, and plucked. The carcass yield was defined as the ratio between the eviscerated-carcass weight and the weight of the feed-deprived bird. To define the eviscerated-carcass weight, the weight of the feed-deprived slaughtered bird without feathers, organs, head, neck, and feet was considered. Soon afterwards, the breast, drumstick, thigh, and wings were cut and weighed. The yield of the cuts was determined in relation to the weight of the eviscerated carcass.

On the 17th, 18th, 19th, 20th, and 21st days of age, 3 mL of samples were collected from the jugular vein of one bird per experimental unit, totaling five birds per treatment. The anticoagulant ethylenediaminetetraacetic acid (EDTA) was used in the proportion of 0.1 mL to 1.0 mL of blood for the blood collection. The following hematological tests were performed: erythrocytes and leukocytes count, using the blood sample and 0.1% Toluidine Blue in a 1:200 dilution, with the count performed (N/mL) in a Neubauer chamber; packed cell volume (PCV), determined by refractometry, using the plasma from the same microhematocrit capillary tube [13]; and hemoglobin concentration, by the cyanmethaemoglobin method [14]. With these results, the following Wintrobe’s [15] RBC indices were calculated by using standardized formulae: mean corpuscular volume (MCV), and mean corpuscular hemoglobin concentration (MCHC).

### Statistical Analysis

All data were analyzed using the mixed-models procedure of SAS (Statistical Analysis System, 9.1).

Data were analyzed using a factorial effects model that included FS inclusion (two levels), CO inclusion (four levels), and all two-way interactions as fixed effects. Orthogonal polynomial contrasts were used to assess the significance of linear or quadratic models to describe the response of the dependent variable to increasing CO levels. When appropriate, means were compared using Tukey’s significant difference with an α level of 0.05.

## Results and Discussion

The average values for maximum and minimum temperatures and relative humidity of the air recorded within the shed in the period from 1 to 7 days were 32.4°C, 25.4°C and 76.93%, respectively. In the period from 8 to 21 days, the observed maximum and minimum temperatures were 33°C and 25°C, respectively, with 78.2% humidity.

No interaction was observed (*p>*.05) between the addition of ferrous sulfate (FS) and the levels of cottonseed oil (CO) for the variables feed intake (FI), weight gain (WG), and feed conversion (FC) in the period from 1 to 7 days of age (Table 3). Feed intake, weight gain, and feed conversion were not influenced (*p>*.05) by the level of inclusion of cottonseed oil in the diet. Regarding the addition of ferrous sulfate, no effect was observed on feed intake (*p>*.05), probably because this compound did not affect the palatability of the diet in the amount added, although some authors have reported a significant decrease in feed intake by layer hens in the period from 24 to 28 weeks as the level of iron in the diets was increased [16].

**Table 3:** Effect of levels of cottonseed oil (CO) with or without addition of ferrous sulfate (FS) on feed intake, weight gain, and feed conversion of broilers from 1 to 7 and 1 to 21 days of age.

Main effect	FI	WG	FC	FI	WG^1^	FC^2^
**CO (%)**	1 to 7 days (Kg)			1 to 21 days (Kg)		
0	0.100	0.094	1.069	1.026	0.686	1.498
2	0.104	0.094	1.118	1.046	0.708	1.481
4	0.103	0.096	1.084	1.067	0.742	1.441
6	0.100	0.093	1.082	1.053	0.729	1.448
FS*						
Without	0.102	0.099A	1.034B	1.058	0.726	1.461
With	0.101	0.089B	1.142A	1.036	0.704	1.473
CV (%)	5.00	5.45	6.90	4.00	5.45	3.40
**Source of variation**	**Probability > F**					
FS	0.853	0.0001	0.0001	0.128	0.136	0.499
CO	0.135	0.652	0.503	0.201	0.007	0.029
CO × FS	0.232	0.904	0.377	0.660	0.398	0.386

FI, feed intake; WG, weight gain; FC, feed conversion; CV, coefficient of variation;

*Means with common uppercase letters in the column do not differ statistically according to the SNK test (*p<*0.05);

^1^Linear effect (WG = 0.690 + 0.008CO, R^2^ = 0.76).

^2^ Linear effect (FC = 1495,5–0.00939CO, R^2^ = 0.82)

Weight gain and feed conversion were impaired by the supplementation of iron (*p <* .05) (Table 3). According to Yang et al. [17], basal diets for broilers without iron addition frequently have greater amounts of iron than the 80 to 120 mg/kg recommended by National Research Council [18]. In this context, additional supplementation of minerals like copper, zinc, manganese, and iron may change the expected performance of animals, since alterations in their basal-diet levels can affect the processes of digestion and absorption in the duodenum, the main site of absorption of nutrients. Considering that very young animals absorb almost the entire iron dose administered [19] and that the formation of free radicals in response to ferrous components in the organism has been suggested as an iron-intoxication mechanism [20], it is assumed that the supplementation of iron in diets for broilers in the pre-starter phase might have been detrimental to the performance of these animals, due to the high iron content in diets with ferrous sulfate (388 mg/kg).

An experiment with broiler chickens aged 1 to 39 days was carried out to evaluate the effects of dietary supplementation of ferrous sulfate on performance, in which the diets were formulated based on corn and soybean, containing 107 mg iron/kg diet, and supplemented with 0, 20, 60, 180, 540, and 1,620 mg of iron. In the obtained results, it was found that weight gain increased with the addition of 20 to 60 mg of Fe/kg to the diet; however, higher the supplementation levels led to an increasing reduction of growth. In this study, the apparent iron requirement was 100 mg/kg diet (80 mg from dietary components and 20 mg from supplemented iron). These results were similar to those found in the present study, indicating that excessive amounts of iron in broiler diets may compromise their performance [21].

Because the responses of the birds fed diets containing 2, 4, and 6% cottonseed oil were similar to those of the birds fed the control diet, formulated with soybean oil, it is assumed that up to 6% cottonseed oil can be included in diets for broilers in the pre-starter phase without impairing the performance of these animals, probably because the free-gossypol level present in the oil utilized and the period of consumption were not sufficient to cause undesirable effects, given that the intensity of its toxic action varies according to these factors [22].

Another factor to be taken into account is the metabolizable energy content of cottonseed oil, which is not similar to that of soybean oil (which has around 8,700 kcal/kg, according to Rostagno et al. [10], mainly because of its gossypol content. The use of soybean acid oil in broiler diets replacing soybean oil in equal amounts resulted in losses in animal performance, attributed to the different amounts of triglycerides to activate the entire bile secretion process and formation of micelles (in the case of soybean acid oil having only free fatty acids) and to the proportion of saturated and unsaturated fatty acids, thereby compromising the absorption of fat [23], which did not occur in this study.

Knowing the toxic effects of gossypol, and considering that the maximum level of 720 mg/kg in the diet with 6% cottonseed oil did not compromise performance, it can be inferred that the correction of the apparent metabolizable energy value of cottonseed oil is sufficient for the adjustment of the isoenergetic diets. Therefore, no supplementation of ferrous sulfate is required to minimize the toxic effect of gossypol.

The broiler pre-starter phase is marked by a process of body development and maturation of the digestive system, and so there is a greater need for high-quality feeds and nutrients. After hatching, animals have nutritional reserves from the residual albumen and yolk contained in the yolk sac, which can ensure its survival in the first days of life until they are adapted to independent feeding. However, they do not meet their nutritional requirements because of the immaturity of the gastrointestinal tract, especially concerning enzyme production. Therefore, the adequate nutrition must be provided during this phase so that the nutritional and energy requirements can be met and so that the animal performance is not negatively affected [24].

With regard to the performance of the birds in the total period (1 to 21 days), no interaction was observed (*p>*.05) between the levels of cottonseed oil and the addition or absence of ferrous sulfate in the diets (Table 3). Supplementation with ferrous sulfate did not influence (*p>*.05) the studied variables; additionally, the fact that the responses of the birds fed diets containing ferrous sulfate were similar to those that did not consume it suggests that there is no need for supplementation in this phase, assuming that the gossypol levels of the diets were not increased to the point of impairing the performance of animals. Similar results were obtained by Moreira et al. [1], who evaluated the substitution of soybean meal for cottonseed meal with and without addition of ferrous sulfate in the feeding of pigs in the initial production phase and found that dietary supplementation of iron did not influence the response of the animals, because the amount of free gossypol in the meal was below 400 ppm. Another hypothesis is that its absorption by the animals was lower in this phase, not reaching the point of causing intoxication capable of reducing the animal performance, as observed from 1 to 7 days.

No influence (*p>*.05) of the levels of cottonseed oil were observed on feed intake in this phase; however, although the diets were isoenergetic, weight gain increased linearly (*p =* .009) according to the following equation: (WG = 0.690 + 0.008CO, R^2^ = 0.76), and feed conversion decreased linearly (*p =* .*029*) according to the following equation: (FC = 1495.5 − 0.00939CO, R^2^ = 0.82). Weight gain increased as the level of cottonseed oil was elevated (Table 3); thus, the addition of cottonseed oil provided an increase in the amount of essential fatty acid and in the use of liposoluble vitamins, which can improve performance. The increase in weight gain and improvement in feed conversion caused by the inclusion of lipids in the broiler diet may be related to their extra-caloric and extra-metabolic effects. These effects are responsible for increasing the availability of dietary nutrients and improving the energy efficiency as a result of the increase in net energy, which in turn is attributed to the low heat increment of the fats, due to their metabolism [25].

The presence of lipids in the duodenum stimulates the release of the hormone cholecystokinin, which provides an increase in the release of pancreatic enzymes such as protease, lipases, and amylases, consequently improving the utilization of nutrients like carbohydrates and proteins. In addition, fats reduce the food passage rate, and thus they remain longer under the action of the digestive enzymes, thereby increasing the processes of digestion and absorption of nutrients, which has a positive influence on the response of the birds [26]. Murakami et al. [27] observed a linear increase in weight gain with subsequent improvement in feed conversion in the initial rearing period of broilers fed diets containing increasing levels of linseed oil in the period from 1 to 42 days of age, which is also explained by the extra-caloric value of the fats.

No interaction (*p>*.*05*) was observed between the cottonseed oil levels and the supplementation or lack of supplementation with ferrous sulfate on any of the carcass or cuts yield variables, or any influence of the levels of the oil or addition or non-addition of ferrous sulfate (*p>*.*05*) (Table 4). This indicates that cottonseed oil can be included at up to 6% in diets for broilers with no need for inclusion of iron salts, since it does not lead to losses in the yields of carcass, breast, drumstick, thigh, or wings of the birds. Similar results for yield of carcass and cuts were obtained by Lara et al. [28] and Duarte et al. [29], who evaluated carcass yield of broilers fed different lipid sources (beef tallow, poultry offal fat, and crude soybean oil) and concluded that these sources did not influence the yield of carcass and cuts or the abdominal fat of the birds. This implies that the costs, the quality of the fats, and the effects that they may have on the performance, yield, and quality of animal carcasses are factors that should be considered when deciding which lipid to utilize in the formulation of broiler diets.

**Table 4:** Yield of carcass and cuts of broilers fed diets containing different levels of cottonseed oil (CO) with and without addition of ferrous sulfate (FS), at 21 days of age.

Main effect	CYLD	BYLD	TYLD	DYLD	WYLD
**CO (%)**	**(%)**				
0	67.64	33.04	13.61	15.98	12.24
2	68.90	32.64	14.23	16.03	12.10
4	68.13	32.93	14.32	16.15	11.81
6	68.23	32.96	14.41	16.02	11.85
**FS**					
Without	68.46	32.89	14.23	15.95	11.91
With	67.98	32.89	14.06	16.13	12.08
CV (%)	1.82	7.63	7.10	3.70	4.40
**Source of variation**	**Probability > F**				
FS	0.227	0.987	0.929	0.361	0.336
CO	0.087	0.749	0.331	0.893	0.134
CO × FS	0.151	0.817	0.859	0.103	0.763

CYLD, carcass yield; BYLD, breast yield; DYLD, drumstick yield; TYLD, thigh yield; WYLD, wing yield; CV, coefficient of variation.

No interaction was observed (*p>*.*05*) between the levels of the oil and addition or absence of ferrous sulfate on the weight of the organs of the broilers at 7 days of age. The relative values of organs and intestine length of the birds in this phase were not influenced (*p>*.05) by the levels of cottonseed oil added to the diet. On the other hand, the addition of ferrous sulfate interfered (*p <* .05) with the weights of heart, liver, and intestine of these animals, which decreased (Table 5).

**Table 5:** Weight of organs and length of the intestine of broilers fed diets containing different levels of cottonseed oil (CO) with and without addition of ferrous sulfate (FS), at 7 days of age.

Main effect	HR	LV	GZ	BU	PA	SP	IN	TLI	LD	LJ	LI
**CO (%)**	(%)							cm			
0	1.13	4.74	10.23	0.23	0.56	0.11	10.85	93.65	18.88	35.75	33.75
2	1.12	4.59	10.66	0.27	0.56	0.10	10.24	91.00	19.12	34.43	33.92
4	1.16	4.88	9.74	0.25	0.57	0.11	11.55	93.25	18.15	31.35	34.90
6	1.12	4.49	10.81	0.24	0.57	0.11	11.79	93.75	19.65	35.40	34.15
**FS**											
Without	1.20A	4.99A	10.57	0.25	0.58	0.11	11.82A	94.40	19.04	35.18	34.28
With	1.06B	4.35B	10.15	0.24	0.55	0.11	10.40B	91.43	18.86	33.29	34.08
CV (%)	14.04	16.15	13.62	19.40	13.47	21.73	13.16	8.07	15.71	9.11	11.10
**Source of variation**	**Probability > F**										
FS	0.008	0.011	0.347	0.225	0.371	0.849	0.004	0.225	0.848	0.265	0.873
CO	0.961	0.645	0.428	0.491	0.937	0.172	0.010	0.767	0.803	0.384	0.902
CO × FS	0.866	0.798	0.841	0.011	0.867	0.357	0.136	0.327	0.913	0.253	0.449

HR, heart; LV, liver; GZ, gizzard; BU, bursa; PA, pancreas; SP, spleen; IN, intestine; TLI, total length of the intestine; LD, length of the duodenum; LJ, length of the jejunum; LI, length of the ileum; CV, coefficient of variation.

It is known that iron is an element of great importance in the body because of the role it plays in the cells, and one of the functions of greatest relevance is the oxygen transport. However, if on the one hand it is a vital element to the cell, on the other hand it may become extremely toxic because of its catalytic power on the formation of free oxygen radicals [30].

The iron-excretion capacity is limited even in overload conditions; therefore, the progressive increase in iron uptake, be it gastrointestinally or parenterally, will certainly lead to the pathological condition of iron overload [31], because excessive accumulation of iron in the tissues may trigger the formation of free radicals and result in progressive tissue damage in many organs [32], leading to cases of liver cirrhosis, arthritis, and heart diseases [30]. In this regard, it has been suggested that iron excess, catalyzing a reaction known as the Fenton reaction, causes an accumulation of oxygen metabolism reactive species. Among these are the hydroxyl radicals (OH^+^), which are highly reactive and capable of causing lipoperoxidation of cell membranes, cell death [33], and lower relative weights of heart, liver, and intestine (main site of iron absorption) in animals at 7 days, which is the phase when animals absorb larger quantities of iron, which declines as they age [19].

At 21 days of age, there was no interaction (*p>*.*05*) between the levels of oil and the addition or absence of ferrous sulfate on the relative weights of the organs. The weight of the organs also was not influenced (*p>*.*05*) by the levels of cottonseed oil utilized to formulate the diets, or by the addition of ferrous sulfate (Table 6).

**Table 6:** Weight of organs and length of the intestine of broilers at 21 days of age fed diets containing different levels of cottonseed oil (CO) with and without addition of ferrous sulfate (FS).

Main effect	HR	LV	GZ	BU	PA	SP	IN	TLI	LD	LJ	LI
**CO (%)**	(%)							cm			
0	0.65	3.25	3.68	0.16	0.31	0.09	6.00	148.7	26.30	58.30	65.40
2	0.63	2.98	3.95	0.18	0.32	0.09	5.69	145.6	25.60	60.40	58.40
4	0.63	2.91	3.83	0.18	0.34	0.08	5.35	135.7	24.10	53.90	57.10
6	0.64	2.96	3.86	0.18	0.29	0.08	5.63	149.4	27.20	60.70	63.00
**FS**											
Without	0.64	2.97	3.90	0.18	0.33	0.08	5.65	146.9	26.10	58.25	61.85
With	0.63	3.08	3.80	0.17	0.30	0.09	5.68	142.7	25.50	58.40	60.10
CV (%)	12.17	14.85	14.07	25.06	23.54	30.05	10.93	9.61	16.14	9.70	12.31
**Source of variation**	**Probability > F**										
FS	0.571	0.468	0.394	0.212	0.221	0.176	0.893	0.347	0.652	0.934	0.466
CO	0.835	0.159	0.756	0.619	0.338	0.847	0.087	0.018	0.349	0.012	0.029
CO × FS	0.774	0.653	0.073	0.714	0.693	0.933	0.530	0.977	0.798	0.943	0.608

HR, heart; LV, liver; GZ, gizzard; BU, bursa; PA, pancreas; SP, spleen; IN, intestine; TLI, total length of the intestine; LD, length of the duodenum; LJ, length of the jejunum; LI, length of the ileum; CV, coefficient of variation.

No interaction was observed (*p>*.*05*) between the levels of the oil and the addition or lack of ferrous sulfate on the blood parameters of the animals at 21 days. Ferrous sulfate addition did not influence (*p>*.*05*) these parameters (Table 7). Cottonseed oil levels did not affect (*p>*.05) the relative weight of organs or the duodenum length in this phase. An effect of cottonseed oil levels was observed on the lengths of total intestine (TLI), jejunum (LJ), and ileum (IL), but the polynomial regression analysis did not indicate a linear or quadratic effect (p>.05) on these variables. However, some authors have stated that the addition of ferrous sulfate to the diet contributes to increasing the hemoglobin content in animals consuming diets containing gossypol [34], which was not observed in this study, perhaps because the normal utilization of iron, through its chelating action, reduces the hemoglobin synthesis. Nevertheless, supplementing the diet with iron may spare the dietary iron so that it does not interfere with the blood parameters, which can even cause an increase in hematocrit, hemoglobin, and mean corpuscular hemoglobin concentration. This fact was observed by Moreira et al. [1] in pigs fed to diets formulated with cottonseed meal supplemented with ferrous sulfate (100 and 200 mg/100 kg diet), which provided a greater availability of iron for hemoglobin production as compared with the control diet, without addition of iron salts.

**Table 7:** Effect of levels of cottonseed oil (CO) with and without ferrous sulfate (FS) on the blood parameters of broilers at 21 days of age.

Main effect	PCV	STP	RBC	HB	MCV	MCHC	LK
**CO (%)**	**(%)**	**(%)**	**(10^3^/μL**)	**(g/dL)**	**(μm^3^**)	**(%)**	**(10^3^/μL**)
0	29.80	3.05	2315	8.31	129.70	27.93	29.00
2	29.10	3.00	2174	7.90	138.55	26.69	26.50
4	30.10	3.20	2211	7.72	138.55	25.58	23.50
6	29.70	3.15	2228	7.77	135.65	26.17	31.00
**FS**							
Without	29.60	3.10	2173	7.80	137.79	26.18	24.75
With	29.75	3.10	2291	8.05	133.45	27.00	30.25
CV (%)	7.03	8.33	16.31	13.97	15.90	14.40	37.85
**Source of variation**	**Probability > F**						
FS	0.823		0.312	0.481	0.529	0.503	0.142
CO	0.751	0.155	0.756	0.556	0.641	0.598	0.306
CO × FS	0.220	0.212	0.828	0.356	0.697	0.716	0.616

PCV = packed cell volume; STP = serum total protein; RBC = red blood cells; HB = hemoglobin; MCV = mean corpuscular volume; MCHC = mean corpuscular hemoglobin concentration; LK = leukocytes; CV = coefficient of variation.

It must be stressed that the use of crude cottonseed oil in broiler diets will depend on variations in the price of this by-product in relation to the other ingredients (corn) and the vegetable oil utilized in the formulation of the diet.

## Conclusions

Inclusion of up to 6% crude cottonseed oil in balanced diets for broilers in the period from 1 to 7 days of age does not impair the performance of these animals. Supplementation of ferrous sulfate to chelate the gossypol in balanced diets for broilers from 1 to 21 days of age is not necessary, and it may impair performance in the period from 1 to 7 days of age.
